# FinnTwin12 Cohort: An Updated Review

**DOI:** 10.1017/thg.2019.83

**Published:** 2019-10-23

**Authors:** Richard J. Rose, Jessica E. Salvatore, Sari Aaltonen, Peter B. Barr, Leonie H. Bogl, Holly A. Byers, Kauko Heikkilä, Tellervo Korhonen, Antti Latvala, Teemu Palviainen, Anu Ranjit, Alyce M. Whipp, Lea Pulkkinen, Danielle M. Dick, Jaakko Kaprio

**Affiliations:** 1 Department of Psychological and Brain Sciences, Indiana University, Bloomington, IN, USA,; 2 Department of Psychology, Virginia Commonwealth University, Richmond, VA, USA,; 3 Institute for Molecular Medicine FIMM, Helsinki, Finland,; 4 Department of Epidemiology, Center for Public Health, Medical University of Vienna, Vienna, Austria,; 5 Institute of Criminology and Legal Policy, University of Helsinki, Helsinki, Finland,; 6 Department of Public Health, University of Helsinki, Helsinki, Finland,; 7 Department of Psychology, University of Jyvaskyla, Jyvaskyla, Finland; 8 Department of Human and Molecular Genetics, Virginia Commonwealth University, Richmond, VA, USA

**Keywords:** Longitudinal twin-family study, two-stage design, diverse phenotypes, alcohol, smoking, physical activity, diet, mental health, twins, genetics, epigenetics, metabolomics

## Abstract

This review offers an update on research conducted with FinnTwin12 (FT12), the youngest of the three Finnish Twin Cohorts. FT12 was designed as a two-stage study. In the first stage, we conducted multiwave questionnaire research enrolling all eligible twins born in Finland during 1983–1987 along with their biological parents. In stage 2, we intensively studied a subset of these twins with in-school assessments at age 12 and semistructured poly-diagnostic interviews at age 14. At baseline, parents of intensively studied twins were administered the adult version of the interview. Laboratory studies with repeat interviews, neuropsychological tests, and collection of DNA were made of intensively studied twins during follow-up in early adulthood. The basic aim of the FT12 study design was to obtain information on individual, familial and school/neighborhood risks for substance use/abuse prior to the onset of regular tobacco and alcohol use and then track trajectories of use and abuse and their consequences into adulthood. But the longitudinal assessments were not narrowly limited to this basic aim, and with multiwave, multirater assessments from ages 11 to 12, the study has created a richly informative data set for analyses of gene–environment interactions of both candidate genes and genomewide measures with measured risk-relevant environments. Because 25 years have elapsed since the start of the study, we are planning a fifth-wave follow-up assessment.

As with the Older Finnish Twin Cohort and the FinnTwin16 Cohort both reviewed this year in Twin Research and Human Genetics, FinnTwin12 (FT12), most recently reviewed in Kaprio (2013), is a population-based longitudinal study. The early-age baseline assessments in FT12 complement those from the older cohort of adult twins who were first studied in 1975, when the youngest cohort members were of age 18. And FT12 shares essential design features with FinnTwin16: sequential enrollment of five consecutive nationwide birth cohorts of twins to yield large samples (>2500 twin-pairs) with age standardization at enrollment, and equally divided into brother–brother, sister–sister, and brother–sister pairs, with like-sex pairs equally divided by zygosity. Appreciative of evidence then emerging that substance use is best understood from a developmental perspective with some behavioral precursors (high levels of novelty-seeking and low levels of harm avoidance) evident in childhood (Rose, 1998), FT12 was designed for baseline assessments at an early age preceding onset of regular exposure to alcohol, tobacco or other substances. The early-age baseline assessments permit prospective study of known and potential behavioral precursors of substance use and abuse. The original proposal was a cohort-sequential design to enroll twin children at ages 8, 10 and 12, collecting teacher, parent and peer ratings using assessment procedures created by Pulkkinen (1987) for her longitudinal study of social behavior in 8-year-old Finnish schoolchildren. Ironically, however, reviewers of that proposed design questioned the necessity of such early-age enrollment, and the design was revised as a longitudinal study of five consecutive birth cohorts from age 11 to 12. Fortunately, that baseline age was not too late for studying precursors, for at that age only 7% of twins and their classmate controls affirmatively answered the question ‘ever having used alcohol with friends without adults around’ (Rose et al., 2003, p. 275).

Subsequent waves of follow-up in FT12 have created a rich dataset of behavioral assessments from multiple sources across three stages of adolescence and into early adulthood. Results permit identification of risk factors predictive of alcohol use, tracking the trajectories of use across adolescence and subsequent development of alcohol-related problems in early adulthood. FT12 has assessed broad developmental aspects of the twins’ lives from preadolescence, through late adolescence and into young adulthood, with assessments of family environment, relationships with cotwin, with parents and with peers, adding measures of school, neighborhood and community environments, and information on health behaviors, pubertal maturation and lifestyle factors. Intensive study of a subset of the FT12 sample begun during the winter–spring of the year in which twins reached age 12, included peer nominations and in-school questionnaire assessments of twins, and a gender-and age-matched, unrelated nontwin classmate control.

Parents of intensively studied twins were interviewed at baseline with the Semi-Structured Assessment for Genetics of Alcohol (SSAGA; Bucholz et al., 1994), and when the twins reached age 14, they were interviewed with the adolescent version of SSAGA. All interviewed twins were administered several neuropsychological tests at 14, and from those in the two youngest birth cohorts (1986–1987), saliva was obtained pre/post the interview for assays of testosterone (Eriksson et al., 2005) and cortisol.

## A Multilevel Developmental Contextual Approach

**Fig. 1.**
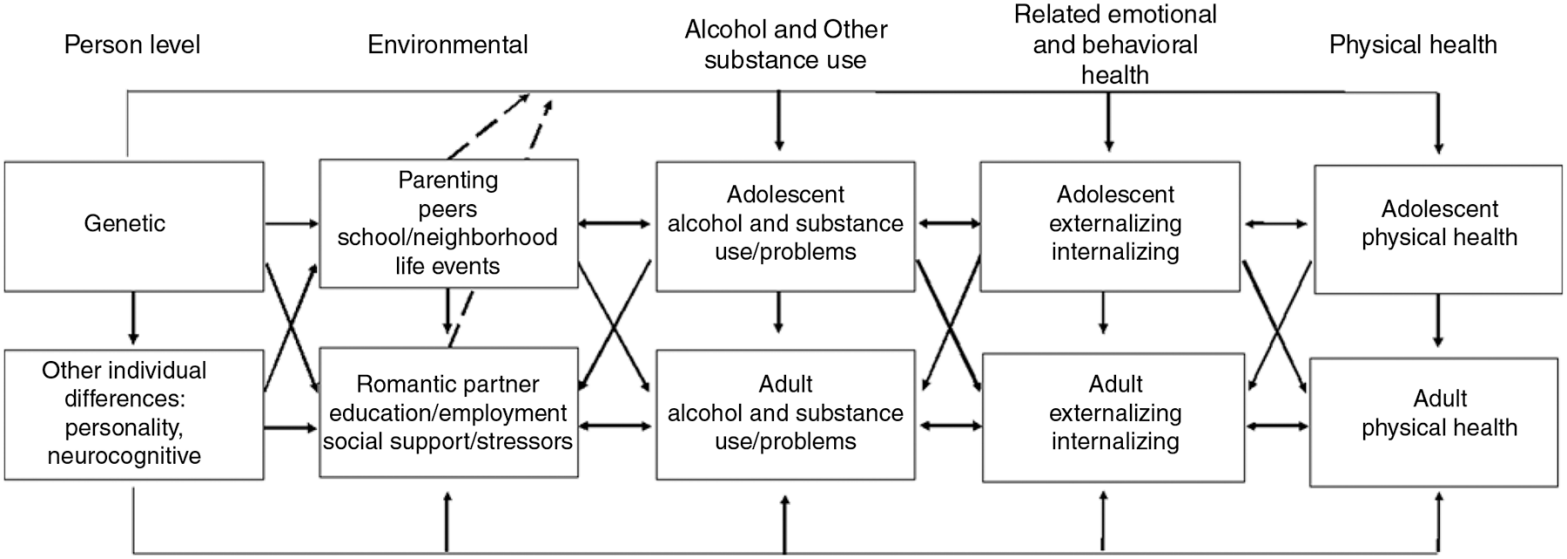
Multilevel developmental contextual framework. Cross paths denote interrelationships among domains. Dashed lines denote gene–environment interaction effects.

Much of the research in FT12 has been guided by a developmental perspective of alcoholism and a multilevel approach to understanding behavioral, emotional and physical health. The diathesis–stress model (Windle, 2010), illustrated in Figure 1, emphasizes the dynamic nature of interactions of individual predispositions and risk factors (i.e., diatheses), environmental risks and buffers (i.e., stressors and protective factors) in initiation and development of substance use and misuse; and other emotional, behavioral, and health outcomes and problems emerging across development. This model recognizes the substantial genetic component underlying these outcomes (Verhulst et al., 2015), as well as the interrelationships between all domains. Analyses of FT12 data offered empirical demonstrations of gene–environment interactions, rooted in the theoretical literature on mechanisms of gene–environment interplay (Dick et al., 2010; Shanahan & Hofer, 2005). The multiple sources of adolescent assessments in FT12, including parallel ratings from twins, their cotwins, parents, peers and teachers, offer prospective study of gender differences in genetic and environmental influences, their correlation and interaction from later adolescence into adulthood.

### FT12 Sample

**Fig. 2.**
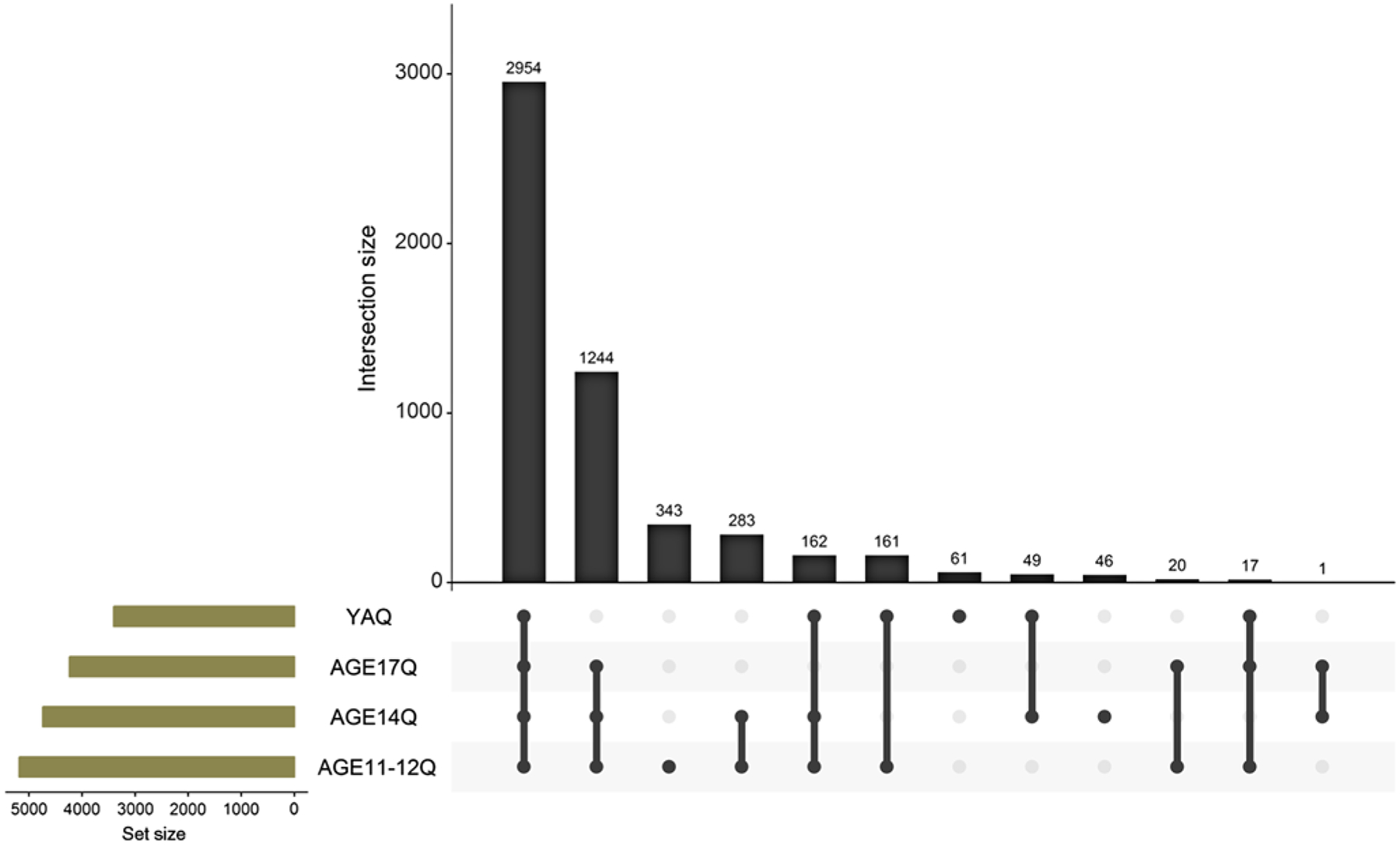
Overlap in responses to wave 1 to 4 studies for the FinnTwin12 twins. The young adult study (YAQ) consists of the intensive sample study (interview, in-person assessments and questionnaires) and the remaining epidemiology sample (questionnaire only).

Twins enrolled into FT12 were born 1983 through 1987; they were identified through Finland’s Central Population Registry (CPR). Ascertainment was exhaustive, and twins in all pairs with both twins alive, resident in Finland and enrolled in normal public schools, were invited to participate. To date, four waves of questionnaire data have been collected from FT12 participants. Key information regarding the participation of the twins and parents in the epidemiological, questionnaire-based study waves is provided in Table 1, while Figure 2 shows the numbers of twins who have replied to all or fewer of the survey waves.

### Stage 1 (Epidemiological) Study

A total of 2705 families (87% of all identified twins in the five birth cohorts, living with one or both biological parents and eligible for study) returned the initial family questionnaire mailed late in the year in which successive twin birth cohorts reached age 11. The family questionnaire was mailed to all families of all eligible twins, using updated residential postal addresses obtained from the Population Registry. Return of the completed family questionnaire gave us permission to contact the twin children, and questionnaires for twin boys and girls, and those for their parents, were then mailed. Parents living apart from their twins were included in these postal mailouts. Parent and teacher ratings of all twins were collected at baseline, with high participation rates; 92% of the parents and 93% of the twins’ classroom teachers returned ratings. Cooperation at these initial stages was not associated with family structure, area of residence within Finland, parental age, or sex or zygosity of twin-pairs. All twins in the FT12 sample were invited to participate in the first follow-up survey at age 14 (with 92% retention). Questionnaires for the second follow-up at age 17 (75% retention) were sent only to those who responded earlier, but in young adulthood (mean age 22, 66% retention), questionnaires were again sent to all (the now adult twins) to permit them to withdraw from further study if they chose to do so.

### Stage 2 (Intensive) Study

Nested within the full epidemiological sample described above is a subset of intensively studied twins (*n* = 1854), for whom we have collected much more detailed phenotypic and genetic information. In creating the invited subsample (1035 families), random sampling preceded selective enrichment for familial alcoholism risk. A random sample (of predetermined *n*) was selected from each birth cohort; to that random sample, all remaining twins in that cohort for whom one or both parents exceeded the threshold on a questionnaire screen for alcoholism (the Malmö-Modified Michigan Alcoholism Screening Test; Mm-MAST; Kristenson & Trell, 1982) were then added. The subsample of twins selected for familial risk comprised 28% of all families selected for intensive study from the five successive birth cohorts. Parents in the intensively studied subsample were interviewed using SSAGA during the year in which their twin children reached age 12. In the early months of that year, twins in the intensive subsample completed an in-school assessment that included a student questionnaire and peer nominations (Pulkkinen et al., 1999) in classrooms across Finland. At follow-up at age 14, twins in the intensive sample were invited to complete an in-person, adolescent version of the SSAGA and a set of neurocognitive tests.

In young adulthood, the intensive sample of twins was invited to in-person testing in Helsinki. This wave 4 data collection in 2006–2009 was the most comprehensive assessment of the twins conducted to date. It included structured psychiatric interviews at an average age of 21.9 (*SD* 0.8, range 21–26) years: adult SSAGA interviews in person or via telephone if a twin could not travel to Helsinki. The in-person testing also included neuropsychological assessments and collection of DNA and serum samples. In total, 73% completed young adult SSAGAs and 1260 individuals provided DNA [526 of whom were monozygotic (MZ) twins]; 812 also completed a laboratory assessment that included a test battery assessing neurocognitive functioning.

### Measures

We highlight below relevant measures collected from the twins across the categories of the theoretical model illustrated in Figure 1.

#### Alcohol and other substance use.

Beginning in midadolescence, twins in the full epidemiological sample responded to survey questions about their frequency of alcohol use and intoxication, as well as their use of tobacco, cannabis and other substances. In follow-up in young adulthood, twins also reported on their alcohol problems, as measured with the Mm-MAST. Intensively studied twins completed an in-school questionnaire in the spring of the year they turned age 12; it included simple yes/no questions on personal experience with alcohol and cigarettes and that of their peers, and at age 14, they were administered the adolescent version of SSAGA in face-to-face format, yielding symptom counts and diagnoses for DSM-IV alcohol dependence and abuse and nicotine dependence. At age 22, the intensive sample provided information about their substance use, abuse and dependence (smoking, alcohol, cannabis and other drug use) based on questionnaires and interviews for comorbid DSM-IV diagnoses (alcohol, illicit drugs, depression, eating disorders and antisocial behavior), while all twins in the full epidemiological sample were asked about their alcohol and tobacco use in questionnaires.

#### Internalizing and externalizing behaviors.

At age 12, teachers, parents and twins in the full FT12 sample reported on twins’ emotional problems, behavioral problems and social adjustment using the Multidimensional Peer Nomination Inventory (MPNI) and teacher ratings (a different teacher, different school setting) were obtained again on the full sample at age 14. Peer nominations were obtained at age 12 for the intensive sample with the MPNI in parallel teacher, parent and peer format (Pulkkinen et al.,1999). For more information on the development and predictive value of the MPNI, see the recent book by Lea Pulkkinen (2017). Twins responded to questions about their depressive symptoms with the Children’s Depression Inventory at ages 12 and 14 (Kovacs, 1991) and the General Behavior Inventory (GBI) at ages 17 and 22 (Depue, 1987). As part of the SSAGA psychiatric interviews, the intensively studied subsample of twins was assessed for depression, anxiety, suicidal behavior, conduct disorder/adult antisocial behavior and oppositional defiant disorder at age 14 and 22.

#### Person-level factors.

In the age 17 follow-up questionnaire, twins completed the Karolinska Scale of Personality (Klinteberg et al., 1986), with scales tapping predispositions to psychopathology. Additional person-level data, including genotyping and neurocognitive functioning, were collected from participants in the intensively studied subsample at wave 4. The neurocognitive functioning battery included measures of executive functioning (Delis et al., 2001; Närhi et al., 1997; Reitan & Wolfson 1993), general cognitive ability (Wechsler, 1997) and working memory (Wechsler, 1987). The intensive sample at age 22 was also asked about adjustment (12-item General Health Questionnaire GHQ-12), personality (NEO-Five Factor Inventory, NEO-FFI), schizotypy (Schizotypal Personality Questionnaire, SPQ) and sense of coherence.

#### Environmental/contextual factors.

In adolescence, twins reported on their recent life events (Salvatore, Meyers et al., 2015), their parents’ knowledge about their plans and activities, how they were spending their money, where and with whom they spent leisure time (Chassin et al.,1993), and their affiliations with deviant peers. In young adulthood, they reported on whether they were involved in a committed romantic partnership; the number of their social support providers and those with whom they could share feelings and concerns (Barr et al., 2017); years of completed education and student status (Barr et al., 2016; Latvala et al., 2011); their employment status and experiences with unemployment and parenthood.

#### Physical health.

At all ages, participants reported on their self-rated overall health and 10 somatic symptoms (e.g., headache, pain, and tension/nervousness; Silventoinen et al., 2007). Information on illnesses was also asked in most detail at age 22 in the intensive sample, including general health (illnesses, body composition, metabolism, and experienced health). Weight and height were asked at all ages. They also reported on lifestyle factors (physical activity and food habits). The twins were tested for taste and smell, and they were asked about chemosensory preferences in the intensive sample at age 22.

#### Genetics, epigenetics and metabolomics.

Genotyping was conducted using the Human670-QuadCustom Illumina BeadChip (Illumina, Inc., San Diego, CA, USA) and DNA methylation using the 450-k Illumina array. A nuclear magnetic resonance (NMR)-based metabolomics panel was used (Bogl et al., 2016). We have also collected DNA samples from some of the nonintensive twins, and Table 2 gives the numbers with genotypes and epigenetic data.

#### Register follow-up.

We update vital status from the CPR as needed, and Table 2 shows the numbers of twins who have died by participation status in the survey waves. Over time, we will be able to examine causes of death by linkage to Statistic Finland Cause-of-Death Register. In wave 4 and earlier, we have not consented the twins for linkage to other medical registers in Finland, but that is an option we will consider in the next wave of data collection. For details on data availability through the National Institute for Health and Welfare Biobank for those participants with biosamples, please see Kaprio et al. (2019) for our review paper on the older Finnish Twin Cohort.

What follows is an overview of major areas of scientific knowledge to which FT12 data analyses have contributed.

## Genetic and Environmental Influences on Alcohol Outcomes

### Analyses of Gene-Environment Correlations and Interactions

FT12, in combination with FinnTwin16, has been pivotal in advancing our understanding of how genetic and environmental influences contribute to alcohol use and related behavioral health challenges across adolescence and into young adulthood. The genetically informative longitudinal data from a population-based sample with high response rates and multiple ratings, including parent, teacher and peer reports, have provided unprecedented opportunity to characterize the etiology of alcohol use and drinking-related problems across the critical developmental period from adolescence to young adulthood. These data have demonstrated important changes in genetic and environmental influences across adolescence. A fundamental finding is that alcohol initiation in early adolescence is largely influenced by shared environmental factors, with little or no evidence of genetic effects (Rose, Dick, Viken, Pulkkinen et al., 2001). But as individuals progress across adolescence, transitioning from initiation and experimentation to more regular patterns of drinking, genetic effects become increasingly important, accounting for nearly 50% of the variance in frequency of alcohol use by age 18. Concurrently, common environmental influences steadily decline in importance across adolescence (Pagan et al., 2006). In contrast to adult alcohol dependence, which shows significant genetic influence (Verhulst et al., 2015), symptoms of alcohol dependence in very early adolescence are largely environmentally influenced, indicating that the etiological factors underlying alcohol problems at early developmental stages are often to be found in environmental experiences.

Our data have also demonstrated dramatic modulation of genetic effects as a function of multiple environmental contexts across adolescence, with genetic influences on substance use assuming greater importance under conditions of reduced parental monitoring (Dick, Viken et al., 2007), high peer deviance (Cooke et al., 2015), more urban settings (Dick, Rose, Viken et al., 2001) and as a function of related sociodemographic factors (Dick, Rose, Viken et al., 2001; Rose, Dick, Viken & Kaprio, 2001). Furthermore, we found that the importance of relevant moderating environmental factors changes across developmental stages (Dick, Pagan, Viken et al., 2007). In young adulthood, genetic influences on alcohol use vary as a function of romantic relationship status and social support networks (Barr et al., 2017). We have extended these studies to include measured polygenetic risk scores, characterizing the mediating pathways by which genetic risk is operating, and the environments that moderate associations of polygenic risk scores with adverse outcomes (Barr et al., 2019; Li et al., 2017; Salvatore et al., 2014; Salvatore et al., 2018; Savage, Salvatore et al., 2018). These studies underscore the important role of multiple environmental contexts in exacerbating or buffering the impact of genetic influences on alcohol use outcomes emerging during early adulthood.

A series of FT12 studies have characterized effects of early pubertal maturation (Dick et al., 2000; Dick, Rose, Pulkkinen et al., 2001; Savage, Rose et al., 2018), behavioral characteristics of peer friendships (Dick, Pagan, Holliday et al., 2007) and the nature of parental influences on initiation and trajectories of substance use (Latendresse et al., 2008, 2009, 2010; Salvatore, Aliev et al., 2015). One finding of these efforts (Dick, Pagan, Holliday et al., 2007; Dick et al., 2000) has been that females may be more strongly affected by environmental influences than are males; and data from peer nominations at age 12 offer evidence of a protective role of social anxiety on trajectories of substance use, with differential effects for males and females (Savage et al., 2016). We briefly review some of that recent work below.

### Sex Differences

The multiple sources of adolescent assessment in FT12 permitted us to demonstrate shared environmental variance in measured characteristics of home environments (reduced parental monitoring and less supportive household atmosphere). Analyses of drinking patterns at age 14 illustrated cooperative social interaction on abstinence and the peer nomination data from FT12 document significant similarities of peer-assessed behavior of twins and their best friends. Behavioral resemblance was greater in the peer assessments of best friends of MZ cotwins, suggesting that adolescent friendship selection is an active process guided, in part, by our genetic dispositions. Patterns of substance use in early adolescence are especially sensitive to both active selection (adolescents seek to befriend those with behaviors and attitudes matching their own), and reciprocal effects of socialization experiences with the selected peers. A robust predictor of drinking/abstaining in adolescence is drinking or smoking friends, and having friends who drink, smoke, use drugs or engage in delinquent behaviors is more predictive of drinking among girls than among boys.

Our data show that girls are more vulnerable to reduced parental modeling, accelerated pubertal maturation and to effects of associating with substance-using peers (Dick, Pagan, Holliday et al., 2007). Recent analyses of FT12 data provide more evidence of gender differences in vulnerability to risk factors and in trajectories of use and abuse into adulthood. Sex differences in patterns of the association of pubertal development with substance use (Savage, Rose et al., 2018) found shared environmental factors contributed to early puberty and heavier substance use for females, and longitudinal effects of differences in pubertal development at age 12 were generally greater among females; a result shown in the association of puberty development at age 12 with symptom counts for alcohol use disorder (AUD) at ages 14 and 22. In contrast, the association of puberty development with illicit drug use was greater for males.

A negative relationship (i.e., a buffering effect) between social anxiety and alcohol dependence was found using ratings of social anxiety from classmate peers at age 12. The protective effect of social anxiety against alcohol problems at age 22 was greater for boys than for girls. But the association of social anxiety at age 12 with smoking frequency at 14 was more strongly negative (more protective) among girls. Meyers et al. (2014) calculated estimates of genetic risk from symptom counts of AUD obtained in interviews of the cotwin (at age 22) and the parents (when twins were 12) and related these genetic risk estimates to twins’ alcohol use across their development. Results showed significant sex differences: Genetic risk for AUD influenced early adolescent alcohol use more in girls than in boys. In parallel analyses of the association with drinking from genetic risk for externalizing behaviors at ages 14 and 17, sibling interaction effects were found. The associations were greater in females with twin brothers, evidence of positive sibling interaction effects. And in a recent analysis of FT12 data, Barr et al. (2019) asked whether associations of genomewide polygenic scores for alcohol consumption predict alcohol misuse, whether those associations are moderated by romantic relationships, and, if so, whether the gene–environment interactions are consistent across sex. Alcohol misuse was assessed by frequency of drinking, frequency of intoxicating and AUD symptom counts in young adulthood. Genomewide polygenic scores predicted all three measures of misuse, and having a romantic relationship negatively influenced each of the three outcomes. But, interestingly and importantly, the protective effect of a romantic relationship on frequent intoxication was found only in males, replicating other evidence that males are more protected from high-risk drinking by involvement in romantic relationships.

Another recent analysis used both teacher and repeated self-report measurements across the FT12 waves of assessment to show that alcohol use behaviors at ages 12 and 14 predicted lower educational achievement at later time points, and those associations remained even after previous achievement and measured confounders were taken into account (Latvala et al., 2014). In another study, the genetics of perceived family environment was evaluated with data from the full FT12 sample at 12, 14, and 17, yielding evidence that additive genetic effects explained increasingly more of the variation across that 5-year period of development while the role of the environment shared by cotwins decreased (Silventoinen et al., 2019).

Jointly, these FT12 studies have importantly contributed to our understanding the interplay of genetic and environmental risk factors for alcohol use and problems across adolescence and into young adulthood. With future follow-up of this informatively rich twin cohort, we are developing research protocols to characterize alcohol use patterns, predictors and consequences into early midlife.

## Studies of Cannabis/Smoking/Snus

### Testing the ‘Gateway’ Hypothesis in Substance Use

FT12 data have contributed insights into debate surrounding the gateway hypothesis of substance use/abuse – the hypothesis that early exposure to ‘milder’, legal substances, such as tobacco and alcohol, leads to use of ‘stronger’, ‘illegal’, more addictive substances, including cannabis. In an initial test of that hypothesis (Korhonen et al., 2008), we explored factors predicting use of cannabis and other illicit drugs (baseline at age 12 with follow-up at ages 14 and 17) in an epidemiological longitudinal study of >4100 twins. The potential predictors were age 12 baseline measures reported by the twins, their parents or teachers. As individual factors, we tested smoking, alcohol use, behavioral and emotional problems; as peer factors: the number of smoking friends and acquaintances with drug experience; and as family factors: parental substance use, socioeconomic status and prenatal exposure to nicotine. When adjusted for within-family confounds, the significant predictors were early smoking onset, drinking to intoxication, having peers with smoking and drug experience, father’s weekly drinking to intoxication and aggressive behavior. Early-onset smoking was the most powerful predictor among individuals and within exposure-discordant twin-pairs, but whether that association is causal remains uncertain. Accordingly, a follow-up study (Huizink et al., 2010) compared two models: (1) one that describes a direct impact of liability to tobacco use on cannabis and other illicit drug use with (2) a model that assumes shared underlying liability for tobacco and illicit drug use. Tobacco and illicit drug use were assessed at age 17. The multivariate model, including a direct impact of the initiation of tobacco use on illicit drug use, provided the best fit to the data, suggesting that liability to initiate smoking directly affects illicit drug use, findings supporting the gateway hypothesis, although shared underlying liabilities cannot be excluded.

To evaluate the role of externalizing behaviors in the pathway from smoking to drug use, we analyzed whether hyperactivity– impulsivity, aggressiveness, and inattention at 12 predict illicit drug use at age 17 independently, or whether their associations with drug use are mediated through cigarette smoking at age 14 (Korhonen et al., 2010). The association of hyperactivity– impulsivity with drug use was mostly mediated through cigarette smoking. Concerning aggressiveness and inattention, consistent evidence on mediation was seen only among boys. Consistently in all models, the direct association of cigarette smoking on drug use was strong and highly significant, while the associations of externalizing problem behaviors with illicit drug use are partially mediated through cigarette smoking. Because both smoking and externalizing behavior appeared to be important risk factors of illicit drug use, we estimated genetic and environmental influences common to externalizing behavior (at age 12), smoking (at age 14) and initiation of drug use (at age 17). Multivariate Cholesky models were fit to data from 737 MZ and 722 dizygotic (DZ) twin-pairs. Heritability of externalizing behavior was 56%, that of smoking initiation/amount 20/32%, and initiation of drug use 27%. In the best-fitting model, common environmental influences explained most of the covariance between externalizing behavior and smoking initiation (69%) and amount (77%). Covariance between smoking initiation/amount and drug use was due to additive genetic (42/22%) and common environmental (58/78%) influences. Half of the covariance between externalizing behavior and drug use was due to shared genetic and half due to the environments shared by cotwins. Our results indicated that early observed externalizing behavior provides significant underlying genetic and environmental influences common to later substance use (Korhonen et al., 2012).

Finally, we examined the role of overlapping genetic and environmental factors in the relationship between early adolescent conduct problems and substance use in young adulthood (Verweij et al., 2016). We found modest to moderate phenotypic correlations between early adolescent conduct disorder symptoms and substance use in young adulthood. In males, the phenotypic correlations of conduct disorder symptoms with all three substance use variables were explained largely by overlapping genetic influences. In females, overlapping shared environmental influences predominantly explained the phenotypic correlation between conduct disorder symptoms and tobacco and cannabis use. Conduct disorder symptoms in early adolescence appear to moderately predict substance use in early adulthood. In males, genetic influences seem to be most important in explaining the relationship between conduct disorder symptoms and substance use, whereas in females, shared environmental influences seem to be most important.

Smokeless tobacco in the form of snus, a moist powdered pouch tobacco held between lips and gums, is legal in Sweden and has become a popular form of early nicotine exposure in Finland, leading us to test the association between snus experimentation in late adolescence and daily cigarette smoking in early adulthood among men. At age 17, we assessed lifetime use of cigarettes and snus with other potential predictors of cigarette smoking status at young adult follow-up. Analyses focused on 375 men who were never smokers at baseline; when adjusted for age, monthly alcohol intoxication, maternal smoking and peer drug use, snus experimentation increased the likelihood of later daily cigarette smoking nearly four-fold. Results (Araneda et al., 2019) cannot confirm a causal association, but they do suggest that snus experimentation might constitute a risk for initiating use of other tobacco or nicotine products.

## Smoking and Internalizing Disorders

Depression and smoking are strongly associated, but the causal nature of their relationship is uncertain. Depression is a major risk factor for suicide and suicidality, but the role of smoking in risk of suicide is equally uncertain. To address these uncertainties, we have examined longitudinal associations between smoking, depression and suicidality using FT12 data. Across the developmental transition from adolescence (age 17) to young adulthood (age 22), we examined bidirectional associations between cigarette smoking and depressive symptoms in nearly 3000 individuals comprising 1154 twin-pairs. At both waves, self-reported depressive symptoms, assessed with the 10-item version of the GBI, and smoking status were analyzed. When adjusted for multiple covariates and baseline depressive symptoms, daily smokers at age 17 had higher depressive symptom scores at age 22 than did never smokers. Similarly, when adjusted for covariates and baseline smoking, a higher GBI score at age 17 was associated with an increased likelihood of being a nondaily smoker or daily smoker at age 22. No associations were found in within-pair analyses, suggesting that the individual-level association is explained by shared familial liabilities (Ranjit et al., 2019).

With FT12 data, we examined whether tobacco use in adolescence is associated with intentional self-injury and suicide ideation in young adulthood among 1330 twins. After adjusting for multiple potential confounders, those who reported early onset of regular tobacco use and daily cigarette smoking had a significantly increased risk for intentional self-injury, such as cutting or burning, at age 22 in comparison to those who had not at all initiated tobacco use. Early-onset tobacco use was associated with suicidal ideation in females but not in males. Within-family analyses among twin-pairs discordant for exposure and outcome controlling for familial confounds showed similar, albeit statistically nonsignificant, associations (Korhonen et al., 2018).

## Consortium Studies of Aggression in Childhood

FT12 data have contributed to the ambitious, multisite/multinational ACTION consortium (Aggression in Children: Unravelling gene–environment interplay to inform Treatment and InterventiON strategies; http://www.action-euproject.eu/). Collaborative and local analyses of aggression using FT12 data have been conducted. The initial consortium publication introduced ACTION’s 11 partners and its main goal: to improve our understanding of the causes of the individual differences in childhood aggression and co-occurring behavioral and emotional problems (Bartels et al., 2018). That report presented a set of correlations between parent and self-ratings of aggression and other co-occurring behaviors across children aged 3–16 years, using data from six cohorts (including FT12; total *N* > 125,600 observations). Correlations were high between aggression and both externalizing (~.5) and internalizing behaviors (~.4) and generally stable across both age and gender. Co-occurrence patterns were largely similar across countries, instruments and raters; teacher ratings of aggression were studied to investigate aggression and co-occurring behaviors in the school setting using FT12 data and three other ACTION cohorts (*N* = 43,356 observations from ages 7 to 14) and other ACTION consortium analyses utilizing FT12’s genetic, epigenetic and biomarker data are underway (Whipp, Vuoksimaa et al., 2019). Additionally, using only FT12 data, another study has examined the ability of ratings of aggression from different informants (parents, teachers, twins themselves and their cotwins) at ages 12 and 14 to predict antisocial personality disorder in young adulthood (Whipp, Korhonen et al., 2019). The main results indicated that aggression, independent of the influence of hyperactivity/impulsivity, can predict later antisocial personality disorder and that information from multiple informants enhanced the predictive associations.

## Individual Differences in Physical Activity

FT12 data have been used to help explain how essential the interplay between genetic and environmental factors is for understanding individual differences in physical activity. Aaltonen et al. (2018) investigated to what extent the psychosocial home environment in childhood and adolescence modifies the genetic influences on leisure-time physical activity in young adulthood. The study found that if twins had grown up in warm and supportive families, genetic factors had a stronger influence on physical activity behavior in young adulthood. Furthermore, FT12 study interests have focused on the association between physical activity and academic performance. A longitudinal study examined the mutual associations between physical activity and academic performance, suggesting that better academic performance in adolescence modestly predicts more frequent physical activity in late adolescence and young adulthood independent of several familial factors (Aaltonen et al., 2016). A recent study examined the ways in which the association between physical activity and academic performance emerges (Aaltonen et al., 2019). The most important finding from this longitudinal study was that the observed associations between physical activity and academic performance from early adolescence to young adulthood partly resulted from both overlapping genetic influences and overlapping familial environmental effects, which, however, vary in magnitude by age.

## Nuclear Magnetic Resonance-Derived Metabolites, Anthropometrics and Diet

FT12 data have also been used to investigate associations between anthropometric and dietary factors with NMR-derived serum metabolites (Bogl et al., 2016), with particular focus on the lipoprotein subclass profile (Bogl et al., 2013; Jelenkovic et al., 2013, 2014). Factor analysis revealed five distinct dietary patterns, among which a pattern high in fat and sucrose (labeled as the ‘Junk food pattern’) was associated with an atherogenic lipoprotein subclass profile. Habitual fish intake, as assessed by questionnaire or the biomarker serum docosahexaenoic acids, was associated with a favorable subclass distribution of very low density lipoprotein and high-density lipoprotein particles that is considered protective of cardiovascular disease (Bogl et al., 2013). Early puberty and shorter adult height were related to higher concentrations of atherogenic lipids and lipoprotein particles, an association that was substantially explained by common genetic effects (Jelenkovic et al., 2013). The twin data further allowed for the quantification of genetic and environmental factors in the etiology of the associations between serum fatty acids and the lipoprotein subclass profile (Jelenkovic et al., 2014). Abdominal obesity, insulin resistance and low-grade inflammation were associated with changes in the serum metab-olome toward reduced cardiometabolic health (Bogl et al., 2016). Twin modeling and findings from obesity-discordant twins suggest that these associations are partly explained by shared genes but also reflect mechanisms independent of genetic liability. In another study, we tested the hypothesis that prenatal hormone transfer from the male to the female cotwin may result in masculinization of females but found no differences in anthropometric, metabolic or reproductive parameters between females from same-sex and opposite-sex DZ twin-pairs (Korsoff et al., 2014).

## Future Directions

At present writing, the twins enrolled in the FT12 cohort, first studied late in their 11th year of life, now range in age from 32 to 36. This early midlife stage (ages 30–40) is an understudied but important period for trajectories of behavioral, emotional and physical health. The prospective data, collected on the FT12 sample, make this population-based twin sample an unusually informative one for follow-up study. In view of the increasing recognition of the decade of the 30’s as an important developmental period of the lifespan (Schulenberg et al., 2015), it is our hope to conduct a wave 5 early midlife assessment with the FT12 sample. Parallel to the four earlier waves of assessment, participants in the epidemiological and intensive samples would be invited to complete online surveys regarding their current health, behavior, lifestyle and social/interpersonal environments. In view of the salience of romantic/marital relationships on health and behavior in adulthood (Kiecolt-Glaser & Wilson, 2017), we also hope to invite spouses/romantic partners of twins to complete a comparable online survey of their own health habits and lifestyle characteristics and perceptions of the relationship quality with the target twin. Twin participants who have not yet been genotyped will be invited to submit a salivary DNA sample. In this way, we hope to use this valuable resource to understand developmental processes related to emotional, behavioral and physical health outcomes as they unfold across the lifespan.

## Figures and Tables

**Table 1.** Questionnaires and interviews of the FinnTwin12 twin cohort in five waves of data collection. At baseline, a family questionnaire was completed by the parents (*n* = 2705 families out of 3136 contacted) about the twins’ birth and childhood and provided consent for the study. In addition, parents replied to a questionnaire about themselves and their own parents and jointly completed a questionnaire on the twins’ behavior

Survey	Wave	Year(s)	Birth years	Individuals invited	Individuals replied	Mean age (*SD*), range	Nunber of girls/women	Number of boys/men	Number of MZ pairs	Number of DZ pairs	Number of SSDZ pairs	Number of OSDZ pairs
Age 11–12 Q	1	1994–1999	1983–1987	5522	5184	11.4 (0.30), 11–12	2564	2620	834	1612	810	802
Age 14 Q	2	1997–2002	1983–1987	5362	4739	14.1 (0.08), 14–15	2380	2359	754	1437	722	715
Age 17 Q	3	2000–2005	1983–1987	4594	4236	17.6 (0.26), 17–19	2191	2045	682	1280	648	632
Young adult Q	4	2004–2012	1983–1987	4824	3404	24.2 (1.65), 19–28	1933	1471	517	837	428	409
Teacher rating at age 12	1	1994–2000	N/A	N/A	4590	11.6 (0.32), 10–13	2278	2312	739	1391	705	686
Teacher ratings at age 14 Q	2	1997–2002	N/A	N/A	3013	14.2 (0.20), 14–16	1542	1471	461	806	412	394
Parents: Parental ratings of twin behavior Q	1	1994–1999	N/A	N/A	2454	N/A	N/A	N/A				
Maternal Q	1	1994–1999	1940–1968	2738	2573	41.1 (4.9), 29–57	2573	N/A				
Paternal Q	1	1994–1999	1923–1967	2636	2310	43.3 (5.5), 29–83	N/A	2310				

Note: MZ, monozygotic; DZ, dizygotic; SSDZ, same-sex dizygotic; OSDZ, opposite-sex dizygotic.

**Table 2.** Number of deaths, and the numbers genotyped or with methylation data by survey wave. The number of pairs genotyped on genomewide array

Survey year	Number of deaths by 1/1/2018	*N* with genotype data	*N* with methylation data	Number of MZ pairs with at least one twin genotyped	Number of DZ pairs with both twins genotyped
Age 12 Q	45	1444	778	331	345
Age 14 Q	33	1420	765	324	334
Age 17 Q	22	1332	729	300	322
Young adult Q	5	1462	788	296	352

Note: MZ, monozygotic; DZ, dizygotic.
